# Racial misconceptions of the theory of evolution predict opposition to the theory and science in general among a sample of Zimbabwean university students

**DOI:** 10.1016/j.heliyon.2023.e16783

**Published:** 2023-05-29

**Authors:** Tadios Chisango, Langtone Maunganidze, Mpikelelo Maseko, Brian Muchena, Spiwe Ncube, Lissa Hombarume, Anesu Aggrey Matanga

**Affiliations:** a Department of Psychology, Midlands State University (MSU), Harare, Zimbabwe; b Department of Resource Management, Midlands State University (MSU), Harare, Zimbabwe; c School of Social Work, Midlands State University, Harare, Zimbabwe

**Keywords:** Theory of evolution, Acceptance of evolution, Acceptance of science, Racial misconceptions, General misconceptions, Spirituality

## Abstract

The theory of evolution has often been misconceived and often associated with racist undertones and insinuations towards Black Africans, who are assumed to be less evolved and thus closer to “apes” genetically than other, presumably more advanced racial groups. In this research was thus tested the hypothesis that misconceptions surrounding Charles Darwin's Theory of Evolution, particularly racial ones, would predict a lack of acceptance of the theory in particular, and the entirety of the science enterprise in general among a sample of Black Zimbabweans. We also tested the impact of spirituality on both acceptance of evolution and science. The hypotheses received support from the findings and they are discussed in line with pedagogy surrounding evolution and science. The findings of the most central importance were that racial misconceptions, general misconceptions and spirituality predicted both acceptance of evolution and science. In turn, the effects of all these exogenous variables on acceptance of science were mediated by lack of acceptance of evolution.

## 1 Introduction

The theory of evolution through natural selection was originally propounded by Charles Darwin in the “Origin of Species” in 1859 but was also developed independently by Alfred Wallace. It has developed in the neo Darwin period into an ever-elegant account for the origins and meaning of all life on Earth, at least in the minds of informed scholars. Richard Dawkins, one of the most prominent of contemporary exponents of Darwin's theory, calls evolution the “greatest show on earth” in his aptly named book, “The Greatest Show on Earth: The evidence for evolution” [1]. In his other work on evolution, “The Ancestor's Tale” (2004) [2] – one of the most voluminous works in existence on the topic- Dawkins writes in reference to Darwin's theory of evolution: “… biology, unlike human history or even physics, already has its grand unifying theory, accepted by all informed practitioners, although in various versions and interpretations (p. 1). Dawkins (2009) [1] further emphatically argues that due to the massive accretions of evidence that have so far accumulated in support of the theory of evolution - converging from an array of scientific disciplines, including paleontology, geology, molecular genetics, and developmental biology - it is better off not viewed as only a theory, but as a “fact”, in the same sense, for example, that London is in the Northern Hemisphere and Pretoria is in the Southern Hemisphere.

Furthermore, the theory of evolution through natural selection is one of the very few scientific ideas that have historically spawned much more than academic interest to capture the interest of laypeople globally. First, this is possibly because the theory struck immediately and boldly right at the heart of the age-old and still ongoing epistemological debates surrounding the purpose and meaning of human existence [3]. By so doing, it squarely pitted the age-old, intuitively appealing creationist doctrines that lent human life a high, spiritual meaning on the one hand, with an intriguingly cold form of scientific rationalism that sees human life as essentially and biologically indistinct from all other forms of life on the other hand. Secondly, widespread interest in the theory might also reflect the fact that Darwin's theory is among a very small set of scientific theories which also include the big bang theory whose precepts, corollaries and implications are usually quite complex but are deceptively easy to grasp, leading people, for example, to a firm but misleading belief that ‘humans evolved from monkeys’.

This resulted in at least two broad outcomes, with myriad cascading effects. The first is that scholars in different fields of study were quick to apply the concepts of Darwinism to other spheres of life, such as the economy, politics, and social spheres, with a ripple of effects. For instance, Economic Darwinism is pivoted around two concepts. One is “economic selection”, akin to natural selection, which supposes that if some adopted economic behaviour is yielding the lowest magnitudes of payoffs, it will have a higher probability of being replaced by any other arbitrary behaviour. The other economically adopted concept is “mutation”, which implies that at any given point in time, any economic behaviour has a certain probability of “mutating” into an arbitrary behavior [4]. The second application, one of Political Darwinism, is more controversial, and one that carries much more ominous implications. It posits that individuals or social groups can attain an advantageous position over others due to some inherent superiority, such as biological supremacy [5]. In a similar vein, Social Darwinism is a 19th Century doctrine advanced by Herbert Spencer, who championed the idea that social order is a product of the natural selection of those individuals best suited to the current social conditions [6]. There is clearly a fine line between Political and Social Darwinism, with both ideologies endorsing the notion that groups that are more advanced - whether genetically, politically, socially, or economically - will naturally conquer and dominate the less advanced ones. As Pressman (2017) [7] notes, such ideologies –seemingly rooted in authentic scientific reasoning and evidence - rose ominously in a complementary fashion with movements which appealed to racial bigots such as eugenics and racially motivated intelligence testing during an era when scientific knowledge was advancing rapidly, and was regarded as irreproachably prestigious and truthful.

### 1.1 The misconceptions about biological evolution

Related to the fact that the premises of Darwin's theory seem deceptively very easy to understand, the second major outcome was that misunderstandings and misconceptions quickly rose, and many of them are still held as fact today. Many of them are peddled on the internet, appearing sometimes even in textbooks (see Dawkins, 2004 [2], for examples), and are held and propagated elsewhere by individuals who erroneously believe that they have a sound understanding of the theory's tenets [8]. Commendably, contemporary neo Darwinists like Dawkins and Al-Shawaf are doing a stern job to try to debunk and clear such misinterpretations and misunderstandings. Regardless, such misconceptions are deep rooted and hugely prevalent; a basic Google search of “misconceptions of theory of evolution” yields quite a lot of them and they also have attracted the attention of numerous scholars e.g. [9, 10].

Important to note, mistaken evolutionary ideas are driven by and simultaneously have far reaching implications on a lot of philosophical and sociocultural issues. Kaloi et al. (2022) [11] aptly argue that although sound knowledge of evolution chips away at the misconceptions that often characterize its comprehension, the relationship between knowledge and acceptance of the theory is often murky, influenced as it is by a litany of other numerous, diverse and complex factors which operate both in formal classroom settings and informal settings in society. These include young earth creationism and intelligent design movements, which have their roots mainly in religion [12] and political ideologies [13]. For the purpose of parsimony, several taxonomies of the causes of denial of evolution have been advanced. They include the ‘proximate’ or ‘primary’ causes which include religious obstacles, as well as inadequate knowledge/misunderstanding of evolutionary theory in particular and science in general. They also include ‘distal’ causes which include poor teaching, low levels of scientific literacy and general anti-intellectualism see Ref. [14].

Among the ‘distal’ causes, teaching is of particular interest to researchers. According to Nehm and Schonfeld (2007) [15], sound teaching of evolutionary biology provides the “missing link” between scientists' understanding of evolution and the general public's ignorance. However, it is unfortunate that, even in highly industrialized nations like the U.S., the teaching of evolution has remained rather low and/or distorted as a result of a complexity of reasons [16], resulting in rates of acceptance of evolution remaining constantly low over the last two decades [17]. Such a trend has been witnessed elsewhere across the globe e.g. Refs. [[18], [19], [20]]. According to Berkman and Plutzer (2011) [21], the topic of evolution is generally avoided by American teachers, and the majority of them tend to be reluctant in advocating it as an explanation for the origins and existence of life on the planet. Additionally, previous research conducted in Brazil has shown that teachers have a generally poor background in their understanding of the topic. Although the teachers insisted that the concepts surrounding it are easy to grasp, they showed glaring deficits in knowledge when specific questions were posed [22]. Moreover, some studies have suggested that high school Biology teachers show similar attitudes towards evolution as do non-science teachers [23], suggesting that exposure to biology material in tertiary education may do little to improve attitudes towards evolution.

### 1.2 The prevalence of the misconceptions

Whatever negatively affects the understanding of evolution, whether the source is in formal classroom settings or informal social settings, vast swathes of research findings have demonstrated that misconceptions surrounding it are very prevalent. Among other scholars, Bishop and Anderson (1986) [24], Wilson (2001) [25], and Wescott and Cunningham (2005) [26] have administered questionnaires that sought to uncover students' misconceptions about science and evolution. Some items that have featured in such questionnaires include the following: (1) “A species evolves because individuals need to”, (2) “New traits within a population appear at random”, (3) “You cannot prove evolution happened”, and (4) “Survival of the fittest’ means only the strongest survive” (see Wescott, & Cunningham, 2005) [26]. Bemoaning their prevalence and entrenchment, and the antievolutionary stance they inevitably lead to, Dawkins (1996, cited in Allmon, 2011, abstract [15]) remarks: “It is almost as if the human brain were specifically designed to misunderstand Darwinism, and to find it hard to believe.”

The misconceptions have been documented among members of the general public e.g. Refs. [27,28], high school students e.g. Refs. [[29], [30], [31], [32]], non-biology undergraduate students e.g. Ref. [33], undergraduate biology students e.g. Refs. [34,35], medical students e.g. Ref. [36], and science teachers e.g., Refs. [37,38]. For instance, Wescott and Cunningham (2005) [28] observe that even among students of introductory biological sciences, the majority hold preconceptions which impede their capacity to understand the content that is presented in class. Dovetailing with the above findings, Pazza, Penteado and Kavalco (2010) [39] found a high prevalence of such misconceptions among Brazilian students attending biological, physical as well as human sciences, albeit in different degrees. In consideration of the ubiquity of such findings, Almquist and Cronin (1988, p. 522) [40] concluded that students’ understanding of biological evolution “is in considerable need of improvement, especially in the areas of the origins of life, the geographical setting of human evolution …, and the theory of natural selection … [and that] there may be limits to what a college education can hope to accomplish on its own”.

Unfortunately, the misconceptions frequently bear a lot of explanatory power for the naïve student, and are thus often very hard to uproot, having been strongly reinforced through socialization in various sections of society [41,42]. “These misconceptions can be self-constructed or taught/learned and based on experience, vernacular terminology, or religion/myth” (Cunningham & Wescott, 2009, p. 2) [9]. There is a glimmer of hope though. In a study by Nehm and Schonfeld (2007) [15], pre-certified secondary biology teachers participated in a 14-week intervention designed to address misconceptions identified by a pre-course instrument, with mixed results. On the positive side, the findings of the intervention indicated a significant decrease in the misconceptions about evolution and the attendant natural selection process. However, the downside was that teachers’ post-course preference positions remained unchanged; the majority of science teachers still preferred that antievolutionary ideas like creationism be taught in school.

### 1.3 Other factors at play besides the misconceptions

The findings of Nehm and Schonfeld (2007) [15] serve to emphasize the point that there are other factors that are at play other than knowledge that dissuade people from accepting evolution, even among some trained experts, some of which have been pointed out above, including creationist beliefs. The majority of existing evidence suggests that all creationist ideologies, whether they are deemed to equate to religiosity or spirituality, constitute a predominant feature in the rejection of evolution. For instance, within the Judeo-Christian Religions, literal interpretations of the Bible are very likely to lead to anti-evolutionary ideas [21,43,44]. As Kaloi et al. (2022) [11] point out, for many individuals who hold such beliefs, learning facts pertaining to evolution usually does not suffice for them to embrace it, as they perceive it as antithetical and a threat to their religion [45]. Such is the hold of the metaphysical beliefs on individuals that authors like Tolman et al. (2020) [46] suggest a deliberate reconciliation between biology and theology. From our perspective, such an approach is intrinsically contradictory, given the bulky evidence in support of Darwin's theory of biological evolution.

### 1.4 The aim and objectives of the present study

In this article, our aim was to ascertain the role of misconceptions (racial and general ones) in the rejection of evolutionary theory in particular and science in general among a sample of Black Zimbabwean students. It was guided by three main specific objectives. Firstly, we wanted to determine if racial misconceptions pertaining to evolution would have a stronger impact than general misconceptions on the participants’ level of acceptance of evolution in particular, and acceptance of science in general. This is in light of the fact that Black Africans are usually victims of such misconceptions, often being likened to “apes”, and hence being perceived as less human than other racial groups. Secondly, we aimed to ascertain if spirituality offers a competing or complementary explanation to the rejection of evolutionary and scientific beliefs. Our third specific objective was to determine if (lack of) acceptance of evolution would mediate the relationship between the exogenous variables in our model (racial misconceptions, general misconceptions and spirituality) and acceptance of science.

### 1.5 Rationale of the study

The focus of the present study, as summed up by the above objectives, is important for a number of reasons. First, spirituality and creationism occupy the centre of African ontology see [47], which may prevent Africans from accepting scientific explanations of the universe, including Darwinian evolution. Therefore, it is important to determine the role of, and the interplay between, spiritual beliefs and misconceptions about biological evolution, in predicting opposition to evolution in particular and science in general among Africans.

First, the misconceptions are likely to be highly prevalent among African populations, due to poor science teaching practices in our African societies where there is poor availability of funding and resources in tertiary institutions of education, very low embrace of science, and an almost immutable entrenchment of ontological positions that favor metaphysical over natural explanations in regards to any issue see [48]. According to Kasomo (2011) [49], spiritual and religious worldviews are intricately woven into almost every aspect of African life. In African cultures, generally there cannot be death, illness, birth, a natural disaster – any event, any process, any phenomenon – that can be explained in purely mechanical ways, without a metaphysical element.

At the same time, the vast majority of African university students would have only read about human evolution in high school in history textbooks written by history authors with a very rudimentary or no understanding of the actual science of it at all. As far as we know, proper evolutionary biology is hardly taught in high school in Zimbabwe and other African countries. In fact, the recent introduction of a New Curriculum in Zimbabwe further buttressed the teaching of creationist ideologies, with topics such as Islam, Hinduism, Judaism and indigenous religions which were not present in the old curriculum now being taught in class in a new subject now called family and religious studies (FRS) [50]. In neighbouring South Africa, which has a much bigger share of scientists than most countries on the African continent, evolutionary biology was only recently introduced in higher education [51]. According to the above authors, the teaching of the subject is nonetheless faced with big obstacles in South African schools, which include “poor content knowledge, essentialist and teleological reasoning, creationist objections and a perceived racist agenda to the teaching of this subject” (abstract). Furthermore, Jurie van den Heever, a South African evolutionary paleontologist and outspoken anti-creationist, is reported to have said that teachers at South African schools are resistant to teaching evolution [52]. Within the whole Social Science Faculty of our university, where the present research was conducted, evolutionary biology is not taught at all, either as a main or elective module. This is despite the fact that is important for the comprehension of related social science subjects such as Evolutionary Psychology and Sociobiology.

Secondly, it is our belief that such erroneous misunderstandings of the theory of evolution steer away some individuals and certain social groups from even wanting or trying to understand the theory, believing that such misconceptions are in fact true and integral to the theory. This may be particularly true of members of social groups who are targets of derogatory beliefs epitomized by the racially related misconceptions, such as Black People and similar groups like Aboriginal Australians. On the contrary, some individuals who hold high racial prejudicial beliefs against outgroups they deem less evolved may in fact be particularly motivated to believe such misconceptions instead, out of a warped desire to spite and inflict harm on them, hence also inadvertently steering themselves away from a good understanding of the theory. Such misconceptions may in fact have more far-reaching effects, which may include steering members of the derogated groups like Africans and some Aboriginal groups from acceptance of the entirety of scientific knowledge. This assumption is core to the rationale of this study. Whereas it may be common and fashionable for Africans and similar social groups to be derogated for failure to keep at par with the rest of the world in modern scientific and related technological advancements, it is not at all obvious that they would immutably remain so if scientific theories about human origins, evolution and development were not so much used to propagate their so-called genetic inferiority.

There is even a keener reason why the above arguments are of particular pertinence to Black Africans continent and people of Black African descent elsewhere. Specifically concerning human evolution, Africa is commonly known as the “cradle for mankind”. First propounded by Richard Leakey, the “Out of Africa” theory postulates that all modern human beings (i.e., Homo sapiens) are descendants of early human beings who inhabited Africa, some of whom later moved out of Africa and populated the globe see Ref. [53]. Viewed in purely scientific terms, this understanding could lead to a rather humbling and unifying stance that all humanity shares the same genetic origin, and hence essentially belongs to the same big family, regardless of the superficial phenotypic differences that actually reflect little genetic variance which is of little biological meaning. Viewed deep at the genetic level, “whatever we may think as observers, the human species is today especially uniform. Taking such genetic variation as the human population does possess, we can measure the fraction that is associated with the regional groupings *we call races* (italics added for emphasis) … and it turns out to be a small percentage of the total: between 6 and 15% depending on how you measure it – much smaller than in many other species where races have been distinguished” [2, p. 415]. On the basis of this small variation in genetic variance between “the regional groupings we call races”, some scholars hence even argue that racial classifications are of no scientific significance, and are a mere social construction [54].

However, such scientific pronouncements are rather far removed from what happens ordinarily in real life. There is usually a wide chasm between what is known scientifically and the knowledge that derives from intuition and socialization. The postulation that Africa is the cradle of humanity actually seems to lead to a racist belief that the ancient migrations of homo sapiens out of Africa led to the emergence of more evolved races out of Africa, leaving modern-day Africans at some level of evolutionary stagnation, hence somehow meaning that Africans and people of African descent are a less human ‘race’ see Ref. [55]. Indeed, Africans and even African Americans of mixed racial parentage are frequently likened to “apes”, even though, when properly understood within the proper confines of the theory of evolution, all human racial groups undistinguishably fall within the species of homo sapiens, and as a species, humans indistinctly fall into the genus of apes which also includes chimpanzees, gorillas, orang utans and gibbons. In this biological taxonomy, there is no a priori or empirical reason to consider any subspecies or species as genetically superior to any other [2]. By the same token, there is no scientific criterion in existence that permits a genetic gradation of members of the human species from being more “apelike” to being more “human like”, whatever that means. Yet this belief, which essentializes Africans as a distinct, “original”, and therefore presumably less evolved ‘race’, might be a significant part of the bedrock for all racist beliefs pertaining to them. Regardless, Africans may by and large come to believe that such misconceptions are in fact central to the tenets of human evolution, hence downgrading them to a deformed or malformed form of humanity, in this way leading them to shun any beliefs in evolution. Furthermore, this may lead them away from believing in science, having considered evolutionary science and by default science itself as an anti-African enterprise. The above arguments form the central premise of the present research.

### 1.6 Conceptual model and hypotheses

**Fig. 1:**
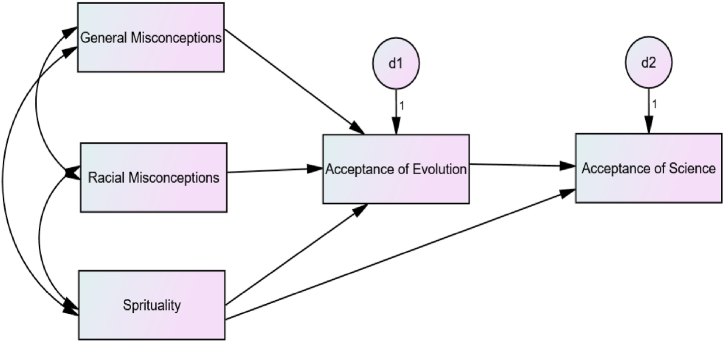
The Conceptual Model Tested in this Research. *Note.* D1 and D2 are disturbance terms.

In light of the above arguments and observations, the current research tests the conceptual model depicted in Fig. 1. In simple terms, the figure depicts the hypothesis that both general and racial misconceptions predict opposition to the theory of evolution by Africans, with racial misconceptions likely to have particularly strong effects. They might believe that the theory carries racist undertones, which feeds in turn into their opposition to science as a mediating variable. Therefore, in this research we draw a distinction between general and racial misconceptions, and test their impact on participants’ acceptance of evolution in particular and science in general. We also included spirituality as another exogenous variable, in addition to the general and racial misconceptions in the model, in order to test its possible unique effects on rejection of evolution and science. In our understanding, the hypotheses encapsulated in the path diagram in Fig. 1 have not been tested in the same model in any of existing research.

Our differentiation of the misconceptions into general versus racial is based upon our knowledge about resistance to evolutionary beliefs among Africans in both lay and academic circles, and also through our reading of related existing literature. In fact, a look at the misconceptions listed by previous authors e.g. Refs. [1,8,28] suggests that they fall into at least two distinct groups: (1) general misconceptions, which reflect commonplace, unmotivated misunderstanding of the theory and (2) racial misconceptions, which imply that some racial groups are “less evolved” than others. The former include the following, among others: (1) ‘a random process like evolution could not produce the complexity and beauty that we see in nature’; (2) ‘evolution is just a theory’ (i.e. implying that it is not backed by any facts or scientific evidence), and (3) ‘evolution is a ladder of progress’, meaning that some species are more evolved than others. The latter include misconceptions such as the following: (1) ‘the Theory of Evolution says some racial groups are more genetically related to monkeys and apes than other racial groups’ and (2) ‘some racial groups are more evolved than others’. Dawkins (2009) [1] scoffs at any such misconceptions as not only wrong, but as deeply antithetical to the notion of evolution itself when it is properly understood.

## 2 Methodology

### 2.1 Participants

A total of 449 students (237 men, 212 women) from the Faculty of Social Science of a university in Zimbabwe, ranging in age from 18 to 23 (Mean age = 21.43, *SD* = 1.29), formed the sample for the study. Informed consent was obtained from all individual adult participants included in the study. The researchers chose students from the Faculty of Social Sciences partly because of convenience, since they all teach in that faculty. More importantly, it was because although evolution is diffusely pertinent in Social Science Fields like Psychology, Sociology, Economics and Anthropology, it is usually not taught at all, but its principles applied, usually crudely, by classroom practitioners with a rudimentary understanding of its tenets. If this is not true universally, this is certainly true of Zimbabwe and many other African countries, where the teaching of evolutionary biology faces a lot of resistance by educators and the society [51,52]. Misconceptions about evolution are thus likely to be quite prevalent in such an educational environment, which gave us an impetus to test our hypotheses. Informed Consent: Informed consent was obtained from all individual adult participants included in the study; no children were involved in the participation of the study.

### 2.2 Procedure

The participants were approached through the E-Learning Platforms and asked to take part in a study on the “Nature of Life and Science”. The questionnaire was uploaded onto the platforms, and only those who were willing to answer it did so. The answered questionnaires were sent back to a research assistant's email account so as to maintain the students' anonymity.

## 3 Materials

The questionnaire comprised five separate scales, which respectively measured the constructs under investigation: (1) general misconceptions (about evolution) (Cronbach's α = . 77, 6 items), (2) racial misconceptions (α = 0.79, 6 items), (3) spirituality (α = 0.80, 5 items), (4) generalized acceptance of evolution evaluation (GAENE) (α = 0.84, 10 items), and (5) acceptance of science (α = 0.85, 10 items). The scales are reproduced in the Appendix.

The acceptance of science scale (BISS) was adapted from Dagnall, Denovan, Drinkwater and Parker (2019) [56] and has been extensively validated e.g. [57, 58]. In turn, the spirituality scale was adapted from Delaney (2003) [59]. The generalized acceptance of evolution evaluation (GAENE) was first developed and validated by Smith, Snyder, and Devereaux (2016) [60]. Furthermore, the general and racial misconceptions were adapted from Wescott and Cunningham (2005) [28] and Dawkins (2009) [1].

### 3.1 Statistical analysis

Preliminary Analysis: Descriptive Statistics, T-tests, Bivariate Correlations and Assessment of Normality.

Descriptive analysis and independent samples *t*-test were used to describe and compare the demographic data (age, gender, and biology study in high school). The correlations between all the measured variables were examined using Spearman correlation coefficients, with use of SPSS 26.0 for Windows. Statistical significance was defined as a two-tailed *p*-value of <.05. Assessment of univariate normality was also conducted of the data, in line with the guidelines set by Kline (2011) [61]. According to their recommendations, skewness values should not exceed the absolute value of three and no kurtosis values exceeded the value of ten (see Table 2).

### 3.2 Main analysis: path analysis

We conducted a path analysis using AMOS 23.0, using the Maximum Likelihood method of estimation. Bootstrapping was performed with 5000 samples to investigate the hypothesized relationships between general misconceptions, racial misconceptions, spirituality, acceptance of evolution and acceptance of science. The mediating effects were determined at the 90% confidence intervals.

## 4 Results

### 4.1 Preliminary results: descriptive statistics, bivariate correlations and assessment of normality

Descriptive statistics and bivariate correlations are presented in Table 1. Racial misconceptions had the highest scores, followed by spirituality and general misconceptions, with acceptance of science and acceptance of evolution having the lowest scores. Table 1 shows that most correlations among the main variables were high and were in the expected directions. For instance, both general and racial misconceptions negatively correlated with acceptance of evolution and science. At the same time, spirituality positively correlated with both types of misconceptions, as was expected.

**Table 1:** Descriptive statistics and correlations among all the measured variables.

			
	*M(SD)*	1	2	3	4	5	6	7
1. General Misconceptions	3.05(0.69)	–						
2. Racial Misconceptions	3.23(0.96)	.41**	–					
3. Spirituality	3.05(0.85)	.58**	.62**	–				
4. Acceptance of Evolution	2.75(0.76)	−.49**	−.69**	−.71**	–			
5. Acceptance of Science	2.90(0.67)	−.29**	−.45**	−.48**	.59**	–		
6. Age	21.43(1.29)	−.01	−.02	−.02	−.08	.09	–	
7. Gender	–	.03	−.01	.02	.00	−.07	−.06	–
8. Biology Major	–	−.03	−.02	−.01	.05	.09	.04	.01

*Note.* **p < .001.

**Table 2:** Normality assessment.

Variable	Skew	*C.R.*	Kurtosis	*C.R.*
General Misconceptions	.292	−2.529	−0.005	−.020
Racial Misconceptions	.542	−4.686	−.573	−2.480
Spirituality	.598	−5.169	−.592	−2.561
Acceptance of Evolution	.274	2.366	−.296	−1.280
Acceptance of Science	.180	1.554	.175	.759

Of the bivariate correlations involving demographic variables, age and gender had non-statistically significant correlations with all the other variables, with only biology study in high school showing a marginally significant correlation with acceptance of science.

Assessment of Normality showed univariate normality in the data, in line with the guidelines set by Kline (2011) [61]. No skewness values exceeded the absolute value of three and no kurtosis values exceeded the value of ten (see Table 2).

### 4.2 Main analysis results

#### 4.2.1 Fit indices

The results of the path analysis are reported in line with Kline's (2016) [62] recommendations, which involve following a three-step process for assessing model fit instead of relying solely on fit indices. According to Stone (2021) [63], fit indices can be easily biased, and are usually cherry picked by researchers to support the apparent fit of their models and the (mis)use of them to justify poorly fitted models. In the face of these dilemmas, Kline (2016) [62] recommends first reporting the exact fit test. If the model passes the exact fit test, then the researcher will provisionally retain the model as one plausible fit for the data. If the model fails the exact fit test, then the researcher will tentatively reject the model. The second step involves assessing standardized and correlational residuals. Kline recommends that researchers reject the model if there are numerous correlational residuals (associated with significant standardized residuals) with an absolute value of greater than 0.1 and retain the model if there are no significant correlational residuals, regardless of the fit indices. This step implies that models that pass the exact fit test but with large correlational residuals may be rejected and at the same time models that fail the exact fit test may be retained if they have low correlational residuals, thus ensuring robustness of interpretation. Step 3 involves reporting RMSEA, CFI, and SRMR but not necessarily using these fit indices to justify the model fit if the first two steps show suggest substantial discrepancies.

The chi-square value was statistically significant, hence initially suggesting poor fit of the model with the observed data: χ 2 = 7.25 (2, N = 795), *p* = .027. However, Ropovik (2015) [64] notes that a statistically significant chi-square value is often ignored on the grounds that it is overly sensitive to large samples such as ours. Following Kline (2016) [62], we further checked if there were numerous correlational residuals with an absolute value of greater than 0.1, in which case we would reject the model. Most of the correlational residues were close to zero, thus leading us to retaining the model.

Last, we report the obtained values of RMSEA, CFI and SRMR, together with their recommended cut-off values, in line with Kline (2016) (see Table 3). According to contemporary standards (e.g. Hair et al., 2014), the accepted thresholds of fit for the above indices are 0.05 for χ^2^, <0.08 for RMSEA, >0.90 for CFI and <0.05 for SRMR. The indices all fell within the accepted thresholds, and taken together, the above results suggest a generally good fit of the model to the observed data.

**Table 3:** Fit indices for the model.

Measure	Name	Obtained Value	Cut-Off Value
CFI	Comparative Fit Index	.994	≥.90
RMSEA	Root Mean Square Error of Approximation	.077	<.08
SRMR	(Standardized) .0Square Root Mean Residual	.020	<.08

#### 4.2.2 Parameter estimates

**Fig. 2:**
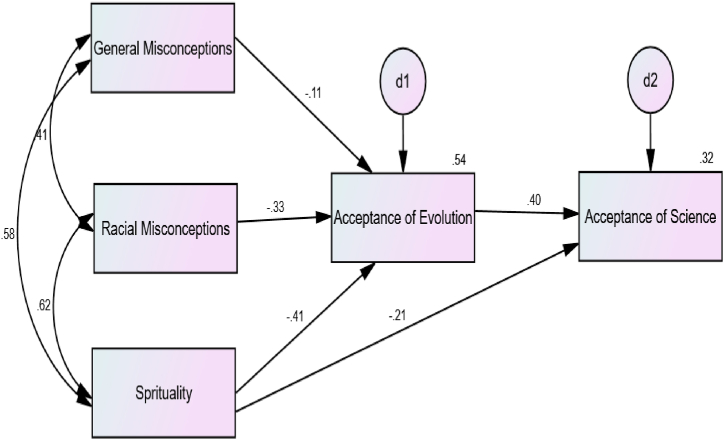
Standardized parameter estimates in the statistical model.

The standardized parameter estimates for this model are presented in Fig. 2. The presentation of the statistics associated with the standard parameter estimates, including the standard errors (SEs), is done in Table 4.

**Table 4:** Output associated with standard regression weights.

		*S.E.*	*C.R.*	*p*
Acceptance of Evolution	<---General Misconceptions	.045	−2.879	.004
Acceptance of Evolution	<---Racial Misconceptions	.034	−8.126	***
Acceptance of Evolution	<---Spirituality	.044	−8.918	***
Acceptance of Science	<---Evolution Belief	.044	7.462	***
Acceptance of Science	<---Spirituality	.042	−4.001	***

Note. ***p < .001.

All the covariances between the pairs of the exogenous variables (general misconceptions, racial misconception and spirituality) were statistically significant, *p* < .001. The direct effects of racial misconceptions on acceptance of evolution and of science were statistically significant (β = −0.33, *p* < .001. The direct effect of general misconceptions on acceptance of evolution was also statistically significant (β = −0.11, *p* < .01). Furthermore, the direct effect of acceptance of evolution on acceptance of science was significant (β = 0.40, *p* < .001), so was the direct effect of spirituality on acceptance of evolution (β = −0.41, *p* < .001) and on acceptance of science (β = −0.21, *p* < .001).

#### 4.2.3 Indirect effects

Importantly, the indirect effect of racial misconceptions on acceptance of science through acceptance of evolution was statistically significant, as evidenced by a 90% bias-corrected bootstrap confidence interval that did not involve zero, indicating mediation. Also, the indirect effect of general misconceptions on acceptance of science through acceptance of evolution was statistically significant, as evidenced as well by a 90% bias-corrected bootstrap confidence interval that did not involve zero, indicating also indicating mediation. Again, spirituality also had a 90% bias-corrected bootstrap confidence interval that did not involve zero, again indicating mediation. See Table 5 for the statistics.

**Table 5:** Bootstrap analysis of indirect effects.

	β	*S.E.*	90% Confidence Interval		*p*
Indirect Effect			Lower	Upper	
General Misconceptions →Acceptance of Evolution → Acceptance of Science		.017	−.070	−.018	.005
Racial Misconceptions →Acceptance of Evolution → Acceptance of Science		.028	−.132	−.061	.001
Spirituality →Acceptance of Evolution → Acceptance of Science		.030	−.168	−.094	.001

## 5 Discussion

First, all the descriptive statistics and correlations were largely of expected magnitudes and/or directions. For instance, as expected, racial misconceptions, spirituality and general misconceptions had high average scores, whereas acceptance of science and acceptance of evolution had relatively lower scores. Bivariate correlations between scores of misconceptions about evolution (both general and racial) and spirituality were also high. In addition, the misconceptions negatively correlated with both acceptance of the theory of evolution and acceptance of science, as expected. Of the demographic variables, only biology study in high school had a marginally significant correlation with acceptance of science.

Second, the path analysis results provided strong evidence to the effect that both racial and general misconceptions in addition to spirituality negatively predict acceptance of the theory of evolution among a sample of Black Zimbabweans. The path analysis results also indicated that the predicted indirect paths were statistically significant, meaning that the paths between, on the one hand, spirituality, racial and general misconceptions and, on the other hand, acceptance of science, were mediated by acceptance of evolution. Important to note, as far as we know, the present research is the first of its kind to provide evidence to the effect that people who reject evolutionary theory also reject science in general. We attribute this finding to the fact that Africans (and other peoples of African descent) are an evolutionarily derogated group, which in turn may lead them to “derogate” and shun the theory of evolution in particular and science in general. These findings however corroborate Lombrozo, Thanukos and Weisbeg's (2008) [65] finding that accepting evolution significantly correlated with understanding the nature of science, even when controlling for the effects of general interest in science and past science education.

As the path coefficient relating to racial misconceptions was much higher than that relating to general misconceptions, the hypothesis that the effect of racial misconceptions would have particularly strong effects was thus supported by the data. However, we suppose that if we had had had the chance to include other racial groups of students in our sample, made difficult however by the fact that our society is largely racially homogenous, the racial differences of racial misconceptions would have been much clearer, with strongest effects likely to be strongest among Black Africans. This calls for further research in much more racially diverse societies.

In general, the above results have far reaching implications, especially on the pedagogy surrounding the theory of evolution in general and science in general. The misunderstandings pertaining to biological evolution are so deeply entrenched and prevalent that a subfield of ‘evolution education’ has emerged among the biological sciences. However, as Hanisch and Eirdosh (2020, p. 2) [66] aptly observe, “evolution education, however, remains largely a disciplinary endeavor of biology education, and educators are left with little guidance on interpreting the broader interdisciplinary applications of modern evolution science discourse”. This has a special implication on social sciences like Psychology, Sociology, Economics and Anthropology where evolution is diffusely pertinent, but is usually not taught at all, whereas its principles are applied, usually crudely, by classroom practitioners with a rudimentary understanding of the evolutionary process. In fact, out of unsound understanding of evolutionary biology, some evolutionary psychologist have made doubtful and strange statements about evolution of human thought and behaviour that have attracted the attention of evolutionary biologists. For example, Smith asks “Is Evolutionary Psychology possible?”: “In this article I argue that evolutionary psychological strategies for making inferences about present-day human psychology are methodologically unsound” (abstract) [67].

The racial misconceptions however have particular conceptual relevance for Africa; therefore they are discussed here at greater length. It is very common, even for highly educated people, to pose questions like “if evolution is true then why have we not seen any monkeys turning into humans?” e.g. Ref. [68]. Such a question is premised upon the false belief that humans evolved from monkeys. This insinuates that actual living monkeys somehow abruptly morphed into humans in ancient times and by the same token modern-day monkeys should morph into humans. Such strange views pertaining to evolution unfortunately are very common, despite being highly implausible, leaving the theory open to high levels of skepticism.

When viewed in conjunction with an equally warped understanding of the Out of Africa theory, such misconceptions lead to the belief that there is an evolutionary scale of progress from monkeys to Africans and then to “more evolved” human races like Whites see Ref. [57]. Whereas such beliefs are known by informed scientists to be deeply antithetical to the notion of evolution, they are not only very common but also deeply ingrained in the minds of many people. Such beliefs may thus serve to bolster racist ideologies among members of racial groups who consider themselves as “more evolved”, whilst driving away racial groups like Black Africans, who are perceived as “less evolved”, from a good understanding of the theory of evolution. Dawkins (2004, p. 4/5) [2] mocks all such kind of thinking:

Biological evolution has no privileged line of descent and no designated end ….there is no reason other than vanity … to designate any one a more privileged or climactic than any other ….More evolved? What can this mean but that evolution is moving in some pre-specified direction?

One major effect of racial misconceptions is that Africans and any people of African descent may shun studying biological sciences in general, and evolutionary biology in particular, especially in the West where interracial boundaries carry a lot of daily social significance. For instance, Graves (2019) [69] writes that in the United States, the professional biological workforce is composed of 69.5% whites, 21.3% Asians and only 3% Blacks. He goes on to note that African Americans compose only 0.3 (or lower) percentage of the workforce specializing in evolutionary biology. According to him, “racism in higher education (specifically evolutionary biology) creates a culturally non-inclusive environment that systematically disadvantages people of non-European descent” (p.1). In fact, Graves himself was the first African American scholar to hold a PhD in evolutionary biology, only as recent as 1988. Although racial sentiments might not play out in African educational systems as in Western countries, African students may nevertheless also shun evolutionary biology and also science in general as a function of the perceived racial undertones in the field, as suggested by our findings.

Due to the fact that all human beings invariably have an acute need to quench existential curiosities, Africans may thus incline more on metaphysical explanations of human origins and existence. This is supported by the present finding that spirituality had both indirect effects (through non-acceptance of evolution) and direct effects on acceptance of science. The metaphysical explanations may in fact provide an easy way out for Africans from perceived racist science. Since spiritual and religious worldviews are intricately woven into almost every aspect of African life (Kasomo, 2011) [51], they may provide a safe haven within which Africans make sense of their bashed humanity. However, whereas such metaphysical ideas may be more appealing and believable than scientific theories like evolution, they may see Africans further stagnated in scientific advancement. African scholars are thus urged to engage more in tackling beliefs that turn Africans away from science. Although science is subject to misuse by some individuals and rogue scientists, science itself has thrown immense light onto the human mind, and is one thing that carries unprecedented potential to lift poor regions of the world like Africa out of poverty and human suffering.

This article ends with a few recommendations. We recommend that well-informed experts teach the theory as a science and not history teachers as if it were an art as is common in African high schools. This would likely go a long way to stem the misconceptions surrounding the topic in African schools and universities. Although our study had an exclusively Zimbabwean sample, virtually all of existing evidence we know of suggests that evolution is widely misunderstood and resisted in Africa (e.g. Refs. [9,53].

It might be also be prudent to have the relevant textbooks carefully perused and censored by science experts prior to publication, lest they continue to unwittingly peddle otherwise dangerous misconceptions. Whether general or racial, results of the present study warn that all such misconceptions have an undesirable effect of making Africans question the veracity of science. In general, we also urge that evolution education be extended beyond the confines of biology, so that educators in related disciplines are better able to interpret the broader interdisciplinary applications of modern evolution discourse cf [66].

At this juncture, it is also important to point that although the racial beliefs that surround the theory of evolution might have had their genesis in Charles Darwin's “Descent of Man”, he himself was to some degree subject to the same misconceptions as his Victorian contemporaries, well before the advent of fields such as molecular genetics that have in modern times given humanity much more precise and accurate knowledge about evolutionary processes. Dawkins (2006) [70] argues “almost everybody in Britain (and many other countries too) would be judged racist by today's standards” (p. 301). As objective and rational as he was in his analysis of nature, we believe he could not have sounded more racist than any well-informed biologist by today's standards. Hence, we recommend that evolutionary theory should be understood not in the original sense in which it was presented by Darwin, but through the lenses of Neo Darwinian evidence in its favor and against some of the preconceptions that Darwin and other earlier scholars might have held. Therefore, scholars are recommended to draw a firm line between the original propositions carried in Darwin's “Origin of Species” and the process of evolution as it is known today, together with the evidence in its support. Last, it is important to point out that our study relied on a sample of students from the Faculty of Social Science, who are usually largely ignorant of the details central to evolution theory. It thus remains to be seen whether similar findings would be reported using a sample of African or similar students who major in pure science subjects. This remains a grey area, since some of the existing research has shown that even biology students tend to hold misconceptions in regards to understanding evolution cf [41].

## Compliance with ethical standards

Ethical approval was granted by the MSU School of Social Work Research Ethics Committee (Reference Number: MSU-SSW- 2023-17).

All procedures performed in studies involving human participants were in accordance with the ethical standards of the Ethics Committee of our Department and with the 1964 Helsinki Declaration and its later amendments or comparable ethical standards.

## Informed Consent

Informed consent was obtained from all individual adult participants included in the study; no children were involved in the participation of the study.

## Funding

The authors have no funding to disclose.

## Author contribution statement

Tadios Chisango: Conceived and designed the experiments; Analyzed and interpreted the data; Contributed reagents, materials, analysis tools or data; Wrote the paper.

Spiwe Ncube; Lissa Hombarume; Brian Muchena; Mpikelelo Maseko; Anesu Aggrey Matanga: Performed the experiments.

Langtone Maunganidze: Conceived and designed the experiments; Wrote the paper.

## Data availability statement

Data associated with this study has been deposited at Figshare: https://doi.org/10.6084/m9.figshare.19501444.v2.

## Declaration of competing interest

The authors declare that they have no known competing financial interests or personal relationships that could have appeared to influence the work reported in this paper.

## Figures and Tables

**Table:** 

1. Evolution is just a theory – it is not supported by any scientific facts.					
2. According to the theory of evolution, individual organisms can evolve during a single life time.					
3. According to the theory of evolution, organisms evolve on purpose while trying to adapt and survive.					
4. Evolution is merely a historical science and therefore cannot be tested or confirmed.					
5. According to the theory of evolution, humans came from monkeys.					
6. If the theory of evolution were true, we would have seen at least some baboons turning into humans.					

**Table:** 

1. The theory of evolution leads inevitably to racism.					
2. All people who believe in biological evolution are racists.					
3. The theory of evolution says Africans are less evolved than other races.					
4. The theory of evolution says Black Africans are a lesser form of human beings than other races.					
5. According to the theory of evolution, Black Africans are closer to apes genetically than are other human races.					
6. According to the theory of evolution, Black Africans are less intelligent than other racial groups.					

**Table:** 

1. I believe in a Higher Power/Universal Intelligence.					
2. My faith in a Higher Power/Universal Intelligence helps me cope during challenges in my life.					
3. I have a relationship with a Higher Power/Universal Intelligence.					
4. My spirituality gives me inner strength.					
5. I see sacredness in everyday life.					

**Table:** 

1. It is important to let people know about how strong the evidence that supports evolution is.					
2. Some parts of evolution theory could be true.					
3. Evolutionary theory applies to all plants and animals, including humans.					
4. People who plan to become biologists need to understand evolution.					
5. I would be willing to argue in favor of evolutionary in a public forum such as a school club, church group, or meeting of public school parents.					
6. Simple organisms such as bacteria change over time.					
7. I would be willing to argue in favor of evolution in a small group of friends.					
8. Evolution is a good explanation of how humans first emerged on the earth.					
9. Evolution is a scientific fact.					
10. Evolution is a good explanation of how new species arise.					

**Table:** 

1. Science provides us with a better understanding of the universe than does religion.					
1. “In a demon-haunted world, science is a candle in the dark.” (Carl Sagan)					
2. We can only rationally believe in what is scientifically provable.					
3. Science tells us everything there is to know about what reality consists of.					
4. All the tasks human beings face are soluble by science.					
5. The scientific method is the only reliable path to knowledge.					
6. The only real kind of knowledge we can have is scientific knowledge.					
7. Science is the most valuable part of human culture.					
8. Science is the most efficient means of attaining truth.					
9. Scientists and science should be given more respect in modern society.					

## APPENDIX

THE MAIN SECTIONS OF THE QUESTIONNAIRE:

Please answer all the following items in the given scales using a scale of 1 = strongly disagree and 5 = strongly agree.

## Appendix ASupplementary data

Questionnaire Evolution Questionnaire Evolution
