# Supplementary material for: Racial misconceptions of the theory of evolution predict opposition to the theory and science in general among a sample of Zimbabwean university students
Source: Heliyon. 2023 May 29;9(6):e16783. doi: 10.1016/j.heliyon.2023.e16783 (PMC10258415; doi:10.1016/j.heliyon.2023.e16783)
Supplement: Questionnaire Evolution [file mmc1.docx]

Department of Psychology

**Research Programme**


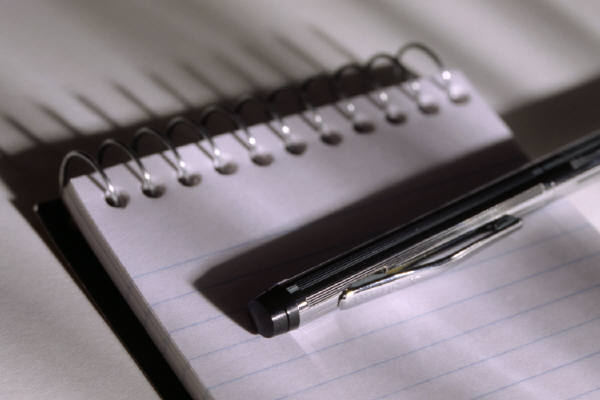


Welcome to our study on the Nature of Life and Science. Please answer fill in the main sections of the questionnaire by marking with an X adjacent to each item using the given scales. Before doing that, please provide your demographic details below.

Age: ____________________

Gender: ____________________________

Please answer all the following items in the given scales using a scale of 1 = strongly disagree and 5 = strongly agree.

|  | Strongly disagree  1 | Mostly disagree  2 | Mostly Agree  3 | Agree  4 | Strongly agree  5 |
| --- | --- | --- | --- | --- | --- |
| 1. Evolution is just a theory – it is not supported by any scientific facts. |  |  |  |  |  |
| 1. According to the theory of evolution, individual organisms can evolve during a single life time. |  |  |  |  |  |
| 1. According to the theory of evolution, organisms evolve on purpose while trying to adapt and survive. |  |  |  |  |  |
| 1. Evolution is merely a historical science and therefore cannot be tested or confirmed. |  |  |  |  |  |
| 1. According to the theory of evolution, humans came from monkeys. |  |  |  |  |  |
| 1. If the theory of evolution were true, we would have seen at least some baboons turning into humans. |  |  |  |  |  |

|  | Strongly disagree  1 | Mostly disagree  2 | Mostly Agree  3 | Agree  4 | Strongly agree  5 |
| --- | --- | --- | --- | --- | --- |
| 1. The theory of evolution leads inevitably to racism. |  |  |  |  |  |
| 1. All people who believe in biological evolution are racists. |  |  |  |  |  |
| 1. The theory of evolution says Africans are less evolved than other races. |  |  |  |  |  |
| 1. The theory of evolution says Black Africans are a lesser form of human beings than other races. |  |  |  |  |  |
| 1. According to the theory of evolution, Black Africans are closer to apes genetically than are other human races. |  |  |  |  |  |
| 1. According to the theory of evolution, Black Africans are less intelligent than other racial groups. |  |  |  |  |  |

|  | Strongly disagree  1 | Mostly disagree  2 | Mostly agree  3 | Agree  4 | Strongly agree  5 |
| --- | --- | --- | --- | --- | --- |
| 1. I believe in a Higher Power/Universal Intelligence. |  |  |  |  |  |
| 1. My faith in a Higher Power/Universal Intelligence helps me cope during challenges in my life. |  |  |  |  |  |
| 1. I have a relationship with a Higher Power/Universal Intelligence. |  |  |  |  |  |
| 1. My spirituality gives me inner strength. |  |  |  |  |  |
| 1. I see sacredness in everyday life. |  |  |  |  |  |

|  | Strongly disagree  1 | Mostly disagree  2 | Mostly agree  3 | Agree  4 | Strongly agree  5 |
| --- | --- | --- | --- | --- | --- |
| 1. It is important to let people know about how strong the evidence that supports evolution is. |  |  |  |  |  |
| 1. Some parts of evolution theory could be true. |  |  |  |  |  |
| 1. Evolutionary theory applies to all plants and animals, including humans. |  |  |  |  |  |
| 1. People who plan to become biologists need to understand evolution. |  |  |  |  |  |
| 1. I would be willing to argue in favor of evolutionary in a public forum such as a school club, church group, or meeting of public school parents. |  |  |  |  |  |
| 1. Simple organisms such as bacteria change over time. |  |  |  |  |  |
| 1. I would be willing to argue in favor of evolution in a small group of friends. |  |  |  |  |  |
| 1. Evolution is a good explanation of how humans first emerged on the earth. |  |  |  |  |  |
| 1. Evolution is a scientific fact. |  |  |  |  |  |
| 1. Evolution is a good explanation of how new species arise. |  |  |  |  |  |

|  | Strongly disagree  1 | Mostly disagree  2 | Mostly agree  3 | Agree  4 | Strongly agree  5 |
| --- | --- | --- | --- | --- | --- |
| 1. Science provides us with a better understanding of the universe than does religion. |  |  |  |  |  |
| 1. “In a demon-haunted world, science is a candle in the dark.” (Carl Sagan) |  |  |  |  |  |
| 1. We can only rationally believe in what is scientiﬁcally provable. |  |  |  |  |  |
| 1. Science tells us everything there is to know about what reality consists of. |  |  |  |  |  |
| 1. All the tasks human beings face are soluble by science. |  |  |  |  |  |
| 1. The scientiﬁc method is the only reliable path to knowledge. |  |  |  |  |  |
| 1. The only real kind of knowledge we can have is scientiﬁc knowledge. |  |  |  |  |  |
| 1. Science is the most valuable part of human culture. |  |  |  |  |  |
| 1. Science is the most efﬁcient means of attaining truth. |  |  |  |  |  |
| 1. Scientists and science should be given more respect in modern society. |  |  |  |  |  |
